# A Case of Suspected Pit Viper Bite in Rural Nepal: Lessons for Snakebite Care

**DOI:** 10.7759/cureus.99292

**Published:** 2025-12-15

**Authors:** Samuel D Horton, Karolina A Szarzanowicz, Nishad Bhatta

**Affiliations:** 1 Department of Emergency Medicine, Sheffield Teaching Hospital, Sheffield, GBR; 2 Division of Medical Education, The University of Manchester, Manchester, GBR; 3 Department of Internal Medicine, B.P. Koirala Institute of Health Sciences, Dharan, NPL

**Keywords:** anti-snake venom, asv, envenoming, haemotoxic, neurotoxic, rural nepal, snake envenoming, syndromic approach, venom-induced consumptive coagulopathy, viper

## Abstract

Pit viper envenoming is common across South Asia. Indian polyvalent antivenom (ASV) does not neutralise pit viper venom. Given the inherent uncertainty in snake species identification, the World Health Organisation (WHO) recommends a syndromic approach to guide ASV use.

A 59-year-old woman was bitten on her right ring finger while cutting grass in the Terai farmland of eastern Nepal. Within one hour, she developed hand and forearm oedema with no bleeding or signs of neurotoxicity. Initial coagulation was normal: the 20-minute whole blood clotting test (20WBCT) clotted, prothrombin time (PT) was 12.4 seconds and international normalised ratio (INR) was 1.0. Urinalysis showed no haematuria. Ten vials of ASV were administered at a peripheral treatment centre with no immediate reaction. She was transferred to a tertiary hospital where she received intravenous (IV) fluids and analgesia and was monitored for three days. No coagulopathy developed on serial testing. The presentation suggested pit viper envenoming, for which ASV is ineffective, so administration of ASV added an avoidable anaphylaxis risk. Snake colour reports can inform, but are insufficient to guide treatment. Management should be syndrome-led, informed by local ecology and based on serial coagulation testing. Clear transfer thresholds and avoidance of bleeding risk practices (intramuscular injections and non-steroidal anti-inflammatory drugs) are vital. System measures, including procurement of antivenom stocks aligned to local snakes, audit, community education and organised transport support early assessment and reduce non-beneficial ASV use. In snakebite where species identification is uncertain, syndromic management guides treatment and reduces iatrogenic harm.

## Introduction

Snakebites are a major cause of morbidity and mortality across South Asia [1,2]. In Nepal, bites cluster with monsoon season agricultural work; pit vipers prevail in hill, mountain and farmland regions, while elapids (e.g., cobras and kraits) and Russell’s vipers can be found in the Terai lowland plains [2]. The main clinical syndromes are haemotoxicity (typically vipers), neurotoxicity (cobras and kraits) and local tissue injury without systemic effects [1,3]. In Nepal, most pit viper bites present with predominantly local effects; clinically significant coagulopathy is rare, although prolonged coagulopathy has been documented after confirmed white‑lipped pit viper envenoming [4].

Haemotoxic envenoming can cause a venom‑induced consumption coagulopathy (VICC). An important initial test to help confirm haemotoxic envenoming is the 20‑minute whole blood clotting test (20WBCT), a simple bedside test where venous blood in a glass tube is left undisturbed for 20 minutes and assessed for clot formation. Results should be complemented by prothrombin time (PT) and international normalised ratio (INR) when available [2,5].

Indian polyvalent antivenom (ASV) is an equine‑derived, polyvalent antibody‑fragment preparation (F(ab’)₂) raised against the “Big Four” venoms. Clinical effectiveness depends on species coverage and timely administration [2,3]. Anaphylaxis is a frequent complication of ASV; intramuscular adrenaline and airway equipment must be immediately available at the bedside, with prophylactic adrenaline given before ASV unless contraindicated (e.g., older patients with suspected ischaemic heart or cerebrovascular disease) [2,6-8].

The World Health Organisation (WHO) and Nepal’s national guidance therefore emphasise a syndromic approach: treat systemic envenoming with ASV when a covered species is plausible; if not, prioritise supportive care and serial assessment, and consider transfer [2,3,9,10]. WHO and Nepal guidance caution that lay colour descriptions are unreliable for species diagnosis. Where possible, identification should rely on expert review of a specimen or photograph, but obtaining these can pose a risk and is difficult to obtain retrospectively. Thus, treatment decisions should remain syndrome‑led [2,3].

## Case presentation

**Figure 1:**
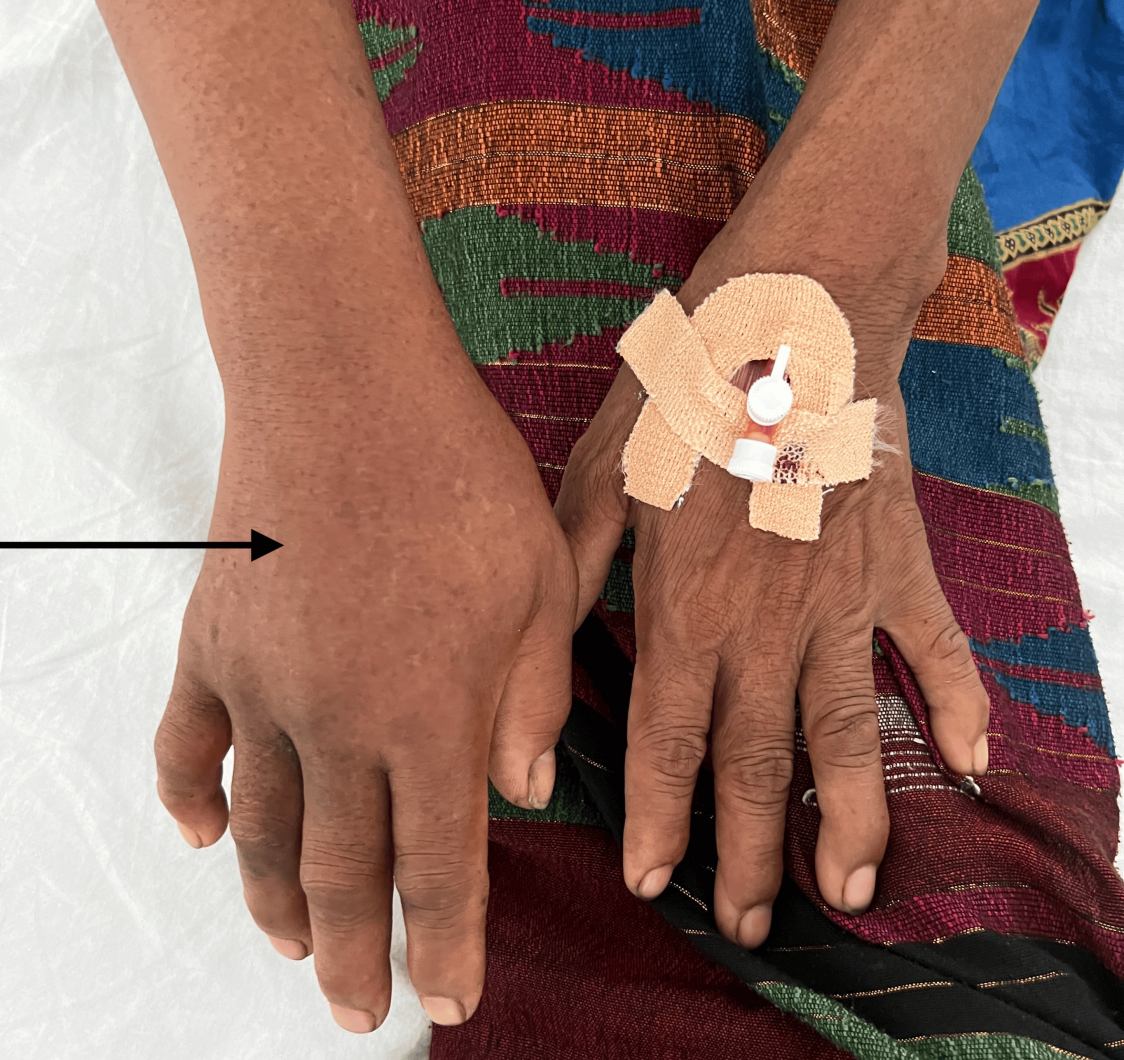
Right hand (arrow) showing oedema extending beyond the wrist following a suspected pit viper bite The left hand (cannulated) is shown for comparison.

A 59-year-old woman with no comorbidities was bitten on the right ring finger while hand-cutting grass in the Terai farmland in eastern Nepal on day 1. She reported a green snake. Within an hour, she developed burning pain and progressive oedema from finger to forearm. There were no initial signs of spontaneous bleeding (e.g., mucosal bleeding, gastrointestinal bleeding or visible haematuria) and no signs of neurotoxicity (e.g., ptosis, bulbar weakness or respiratory involvement). Later on day 1, she reached a peripheral treatment centre. Examination showed two puncture marks on the right ring finger, with marked oedema of the right hand and forearm; the contralateral hand is shown for comparison (Figure 1).

Laboratory findings at presentation are summarised in Table 1.

**Table 1:** Laboratory findings at presentation 20WBCT: 20-minute whole blood clotting test, PT: prothrombin time, INR: international normalised ratio

Parameter	Patient result	Reference range
20WBCT	Clotted at 20 minutes	Clotting within 20 minutes = normal
PT	12.4 seconds	11.0-13.5 seconds
INR	1.0	0.8-1.2
Platelets	233	150-400 × 10^9^/L

Ten vials of ASV were infused; no immediate reaction occurred. She also received intravenous (IV) crystalloid fluids, analgesia, limb elevation and tetanus prophylaxis. She was transferred later on day 1 to a tertiary teaching hospital in eastern Nepal. On arrival, she remained stable and non-neurotoxic. Repeat coagulation tests, including 20WBCT, remained normal. Renal function, electrolytes and venous blood gases were within normal limits. She was monitored from day 1 to day 3 on the acute medical unit. She remained stable with no coagulopathy or neurotoxicity and was discharged home on day 4 with improving swelling.

## Discussion

Indian polyvalent antivenom (ASV) is effective in neutralising the venom of most venomous snakes found in India and Nepal, known as the “Big Four”: the spectacled cobra (*Naja naja*), common krait (*Bungarus caeruleus*), Russell’s viper (*Daboia russelii*) and saw‑scaled viper (*Echis carinatus*).

Although coagulopathy has occasionally been reported after white-lipped pit viper (*Trimeresurus albolabris*) bites [4], the normal 20WBCT, PT and INR in this patient indicated a low likelihood of systemic envenoming. As ASV does not neutralise pit viper venom, its use in this context exposed the patient to risk without a realistic prospect of benefit. Supportive care with serial examination and repeat coagulation testing would have been safer and consistent with guideline recommendations.

**Figure 2:**
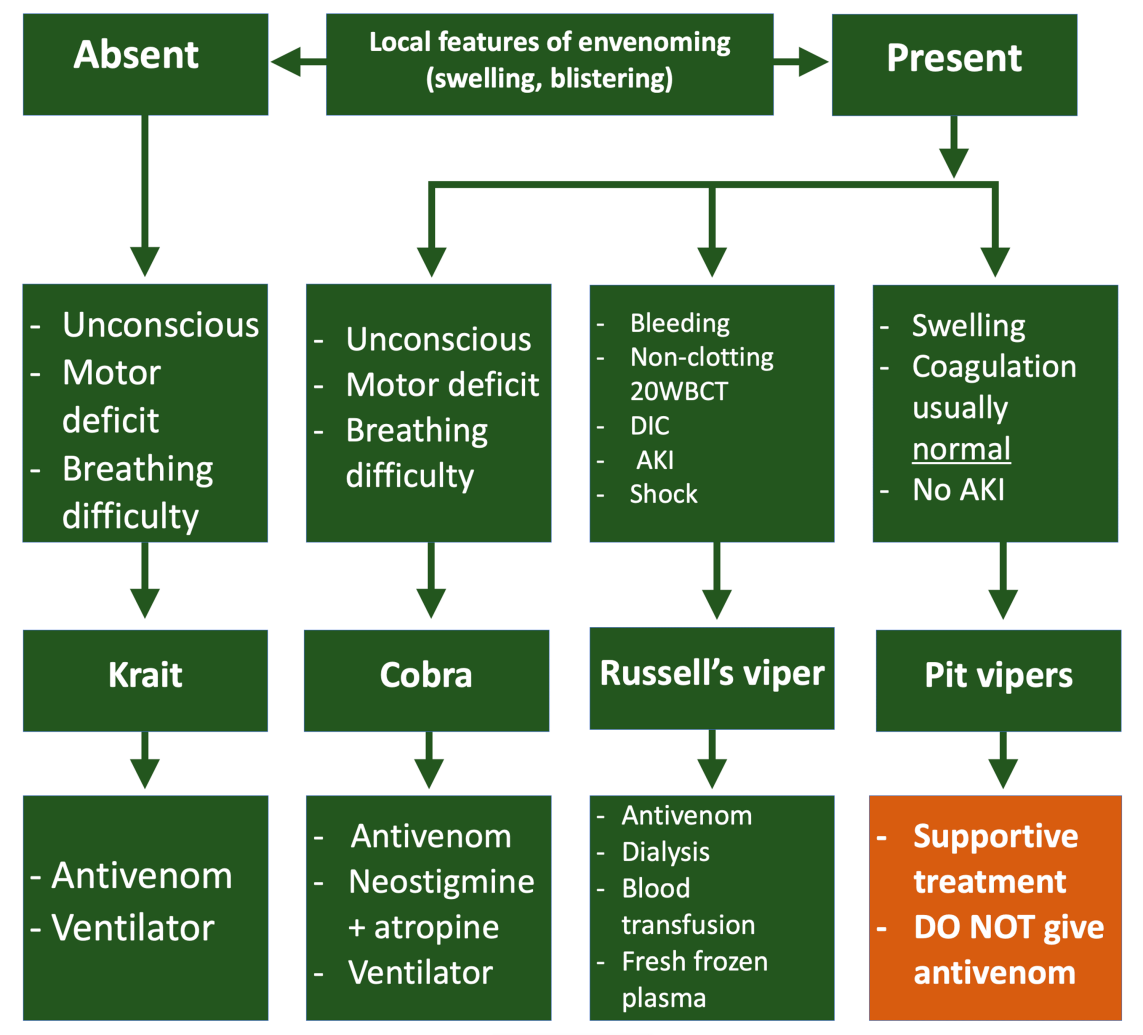
Flowchart outlining a basic syndromic approach to snakebite management in Nepal 20WBCT: 20-minute whole blood clotting test, DIC: disseminated intravascular coagulation, AKI: acute kidney injury Based on the Nepal Ministry of Health, National Guideline for Snakebite Management in Nepal (2019)

To aid decisions under diagnostic uncertainty, the WHO Guidelines for the Management of Snakebites outline a syndromic pathway [2], depicted as a flowchart in Figure 2.

As per the syndromic approach (Figure 2), the significant swelling in the absence of neurotoxicity and coagulopathy indicated that she was likely bitten by a pit viper. The management of this case deviated from the national guidance because ASV was administered when coverage was unlikely [2,3]. The WHO‑endorsed syndromic management recommends ASV only when systemic envenoming is present and ASV coverage is plausible [2]. The clinical picture was of local effects only with normal coagulation: no haematuria, a clotting 20WBCT, normal PT/INR and no neurotoxicity, thus most consistent with pit viper envenoming. The patient‑reported “green” snake was supportive but should not, on its own, guide treatment [2,3]. Furthermore, observation without development of coagulopathy is typical of most pit viper bites. They generally present with local effects only; thus, supportive care with serial testing is appropriate [3,11].

The use of ASV requires careful risk‑benefit consideration given the high rate of acute reactions. In a Sri Lankan randomised controlled trial, 61% of patients developed reactions, with 33% classified as anaphylaxis [12]. In this case from Nepal, administering ASV without plausible coverage exposed the patient to substantial iatrogenic risk with little prospect of benefit. The absence of a reaction does not imply benefit, nor does it justify off‑pathway use.

Where coverage is plausible, early ASV can be life‑saving [2]. Where coverage is unlikely, supportive care is safer [2,3]. Serial PT/INR provides a baseline for escalation and de‑escalation, complementing the 20WBCT and allowing teams to identify emergent or worsening coagulopathy or bleeding [2,3,5]. In peripheral facilities with limited monitoring, predefined triggers (e.g., persistently non‑clotting 20WBCT, rising INR, new bleeding or haemodynamic instability) can aid transfer decisions [2,3].

At presentation, simple changes can make a big difference: ensure the 20WBCT is performed in a clean, dry glass tube [2,5], obtain PT/INR to establish baseline and track coagulation [2] and avoid unnecessary intramuscular injections and non‑steroidal anti‑inflammatory drugs (NSAIDs) in patients at bleeding risk [2]. Then, when ASV is indicated, it should be given by slow intravenous infusion [12] with prophylactic adrenaline [6] (unless contraindicated), under close observation [7,8]. These steps help to reduce predictable hazards in snakebite care and suspected haemotoxic envenoming.

Snakebite outcomes depend on systems as much as bedside care. In Nepal, community education and organised rapid transport shorten pre‑hospital delay and are associated with lower mortality [1]. Regional plans aim to accelerate these gains by 2030 [9,10]. In the eastern Terai, Sharma et al. reported that a multi‑stakeholder awareness programme (village sessions, female health volunteers, posters/leaflets and engagement of traditional healers) combined with a volunteer motorcycle transport scheme was associated with faster presentation and referral. It found a reduction in case fatality from 10.5% to 0.5% and a fall in incidence from 502 to 315 per 100,000 in targeted villages, with no change in control villages [1]. These findings are consistent with the WHO strategy and national guidance, which advise locally tailored education, promotion of early care‑seeking and structured referral and transport networks [1,9,10].

Procurement and referral pathways should reflect local ecology [3,10]. Stocking antivenom products that match regional species reduces unnecessary and potentially harmful exposure and preserves supplies for covered syndromes [3,10]. Seasonal surveillance, feedback from referral centres and regular review of bite patterns help maintain stock. Early presentation should be encouraged, and serial coagulation testing performed as soon as possible to help guide decision‑making [1,9,10].

Regular audit helps assess and improve the safety and effectiveness of ASV use in local hospitals. Research has identified the following metrics: proportion of 20WBCTs performed [2,5], availability and use of same‑day PT/INR in suspected VICC [2], documentation of coverage plausibility before ASV is given [2,3], documentation that prophylactic adrenaline was given or contraindicated [7,8], bedside availability of adrenaline at time of administration [6] and recorded reaction rates to ASV [6].

The case highlights a training gap in services that is modifiable. Focused, case‑based teaching on the syndromic approach [2,3] and correct 20WBCT technique [5] would reduce non‑beneficial use of ASV. Preparedness for anaphylaxis further improves safety when antivenom is indicated [2,6]. Iterative review against the discussed measures helps ensure a syndrome‑led approach, preserves ASV supplies and reduces unnecessary morbidity and mortality from suboptimal treatment.

## Conclusions

Treat syndromes, not species, reserving ASV for envenoming when coverage is plausible. In suspected pit viper envenoming with local‑only syndrome and normal coagulation, ASV should be avoided; supportive care, serial coagulation, and early transfer when appropriate must be prioritised instead. Use 20WBCT in clean glass as the first‑line bedside test for VICC and complement with PT/INR when available. Early anaphylaxis with adrenaline prophylaxis (if indicated) should be anticipated when ASV is given, with adrenaline kept readily available at the bedside. Targeted, case‑based education for regional services on the ASV guidelines should be provided, integrated with community education, rapid transfer programmes and regular closed‑loop audit.
